# Immature Stages and Hosts of Two Plesiomorphic, Antillean Genera of Membracidae (Hemiptera) and a new species of *Antillotolania* from Puerto Rico

**DOI:** 10.3897/zookeys.301.4234

**Published:** 2013-05-17

**Authors:** Stuart H. McKamey, Brent V. Brodbeck

**Affiliations:** 1 USDA/ARS Systematic Entomology Laboratory, 10th St. & Constitution Ave., PO Box 37012, Washington, DC 20013-7012; 2 North Florida Research and Education Center-Quincy, 155 Research Road, Quincy, FL 32351

**Keywords:** Caribbean, Antilles, new species, immature stages, host plant

## Abstract

The nymphs of *Antillotolania* Ramos and *Deiroderes* Ramos are described for the first time, along with the first host record for the genus *Antillotolania*, represented by *Antillotolania myricae*, **sp. n.** Nymphal features of both genera, such as a ventrally fused, cylindrical tergum IX (anal tube), the presence of abdominal lamellae, and heads with foliaceous ventrolateral lobes confirm their placement in Membracidae and are consistent with phylogenetic analyses placing them in Stegaspidinae but in conflict with a cladistic analysis showing a closer relationship to Nicomiinae. Head processes and emarginate forewing pads in the last instars of both genera support an earlier estimate, based on nuclear genes, that the two genera form a monophyletic group in Stegaspidinae. Distinguishing features of the four species of *Antillotolania* are tabulated.

## Introduction

Treehoppers of the family Membracidae are best known for an enlarged, often extravagant pronotum expanded posteriorly over the scutellum (completely or partially concealing the scutellum) or more usually over the entire body. Indeed, until recently this was a diagnostic feature of the family Membracidae. Deitz (1985) was the first since 1928 to include species in this family with pronota that did not project posteriorly – *Hemicentrus* Melichar (Leptocentrini) of the Old World and *Abelus* Stål (Abelini) of the New World. Both *Hemicentrus* and *Abelus* have a distinctly emarginate scutellum, which was characteristic of all membracids that have the pronotum expanded over, but not concealing the scutellum (extant Stegaspidinae and Centrotinae). The emarginate scutellum therefore suggests that the posteriorly projecting pronotum was secondarily lost in *Hemicentrus* and *Abelus*.

Other treehoppers lacking a posteriorly projecting pronotum, but with an acuminate or truncate scutellum, were placed in the treehopper families Aetalionidae, Biturritiidae, and Nicomiidae (Metcalf and Wade 1965). Based on a the first phylogenetic analysis of the superfamily membracoidea (Dietrich and Deitz 1993), Deitz and Dietrich (1993) referred some nicomiid species to the treehopper family Aetalionidae but incorporated many of these taxa into a newly defined Membracidae as the subfamilies Endoiastinae and Nicomiinae, except for two genera for which they erected the new family Melizoderidae. They left four genera, previously placed in Nicomiidae (with short pronotum) unplaced within Membracidae: *Holdgatiella* Evans, *Euwalkeria* Goding, *Antillotolania* Ramos, and *Deiroderes* Ramos.

*Antillotolania* and *Deiroderes* (Ramos 1957) are endemic to the northern Antilles. There are also a number of membracids without a posteriorly projecting pronotum known from Eocene-Miocene amber deposits from the Dominican Republic (McKamey 1998); none have been described but one was correctly identified (Shcherbakov 1992) as a member of the subfamily Stegaspidinae.

Several attempts have been made to determine the phylogenetic placement of *Deiroderes* and *Antillotolania* within Membracidae. In a molecular phylogenetic investigation of Membracidae, Cryan et al. (2000) found these two genera to be placed with Microcentrini (Stegaspidinae), although the subfamily was paraphyletic in that analysis. Dietrich et al. (2001) recovered, in a cladistic analysis of Membracidae based on morphological evidence, *Deiroderes* within the subfamily Stegaspidinae, whereas *Antillotolania* was placed as the sister group to (Nicomiinae + (Centronodinae + Centrodontinae)); statistical support for those placements was equivocal, however.

In a separate phylogenetic analysis based on morphological evidence, Cryan et al. (2003) recovered [*Deiroderes* + *Antillotolania*] as the monophyletic sister-group to [Microcentrini + Stegaspidini]. Later, Cryan et al. (2004) presented the results of an analysis combining molecular and morphological evidence, yielding similar placements of *Deiroderes* and *Antillotolania* as in the Cryan et al. (2000) study; they concluded that both *Deiroderes* and *Antillotolania* should remain unplaced within Stegaspidinae until further analysis could resolve these relationships.

Cryan and Bartlett (2002) described two new species of *Antillotolania* but left the genus unplaced, noting conflicting hypotheses of relationship between the Dietrich et al. (2001) morphological analysis, which suggested it was allied to Nicomiinae, and that of Cryan et al. (2000), in which *Antillotolania* was most closely related to *Deiroderes* and some Stegaspidinae. They suggested that it may be warranted to expand the concept of Stegaspidinae to include both *Antillotolania* and *Deiroderes*. Cryan and Deitz (2002) described a new species of *Deiroderes* and a new genus, *Togotolania*, also from the Antilles that lacks a posteriorly projecting pronotum. They referred *Deiroderes* to unplaced Stegaspidinae and argued that their new genus most likely is allied to Nicomiinae.

Both cladistic estimates incorporating morphology (Dietrich et al. 2001, Cryan et al. 2004) used features of immatures, hitherto unknown for *Antillotolania* and *Deiroderes*. Both genera are exceedingly rare in collections and no immatures were known.

In the present paper we describe a new species of *Antillotolania*, with host and habitat based on multiple series of adults and immatures collected along the central mountain range of Puerto Rico and describe its immature stages. We also describe the fifth instar of *Deiroderes*, based on one specimen collected from the reported host and adjacent to the type locality of *Deiroderes inermis* Ramos in the xeric region of Guánica, Puerto Rico. We also discuss the subfamiy placement of the two genera in the light of the new evidence.

## Taxonomy

### *Antillotolania* Ramos

Prior to this work, this genus contained three species: *Antillotolania doramariae* Ramos and *Antillotolania extrema* Cryan & Bartlett from Puerto Rico, and *Antillotolania microcentroides* Cryan & Bartlett from Guadeloupe and Tortola (British West Indies). These are represented by a total of seven specimens and nothing is known of their biology. No male of *Antillotolania extrema* has been collected. The new species is represented by 11 adult specimens and nymphs.

The originally monotypic genus was described based on one female, lost, and one male, both from Maricao, Puerto Rico. The forewing venation, which contains phylogenetically important characters, differed in the two illustrations. In recent years, a few additional *Antillotolania* have been captured by sweeping vegetation in the U.S. Virgin Islands (J. Cryan, C. Bartlett, pers. comm.), which has enabled their incorporation into phylogenetic estimates using molecular data (Cryan et al. 2000; Cryan et al. 2004).

#### Antillotolania myricae

McKamey & Brodbeck sp. n.

urn:lsid:zoobank.org:act:9588EEE0-5578-4335-8EAD-282468C3434E

http://species-id.net/wiki/Antillotolania_myricae

Figs 1 -17 , 28–31

##### Description.

Dimensions (mm): Length with forewings in repose female 6.2, male 5.8, width between humeral angles female 1.8, male 1.6. Head and thorax densely pilose. Head quadrate in anterior view, in dorsal view with two subtriangular projections, longitudinally carinate behind eyes. Forewing (Fig. 9) M and Cu fused at base, 3 m-cu crossveins, 2 r-m veins, R branched into R_1-3_ and R_4+5_ basad of fork of vein M. Hindwing (Fig. 10) with 1 r-m crossvein and 1 m-cu crossvein, cubital vein un-branched, anal vein branched. Pro- and mesothoracic legs lacking cucullate setae. Metathoracic tibia with cucullate setae in rows I, II, and III as follows: ca. 20 in row I along entire length; ca. 10 in row III throughout distal half; and fewer than 10, larger cucullate setae in row II irregularly spaced in conjunction with darkly pigmented sections of tibiae (pale row II edge densely pilose but setae without cucullate bases). Abdomen lacking abdominal lamellae, vestiture (see Dietrich 1989) consisting of microtrichia (Fig. 17), as in *Microcentrus* Stål.

Male (Figs 4–8): Pronotum with small shelf like suprahumeral developments, little more than carinae that do not extend from the pronotal surface (Fig. 4–6). Pygofer and subgenital plates bare, not setose, lacking projections. Styles with base long and subparallel, acute apices recurved laterally (Fig. 7). Aedeagus asymmetrical in dorsal view, lobe of apex curving to the right (Fig. 7); shaft sinuate, directed dorsally then posteriorly, apex expanded (Fig. 8).

**Figures 1–10.**
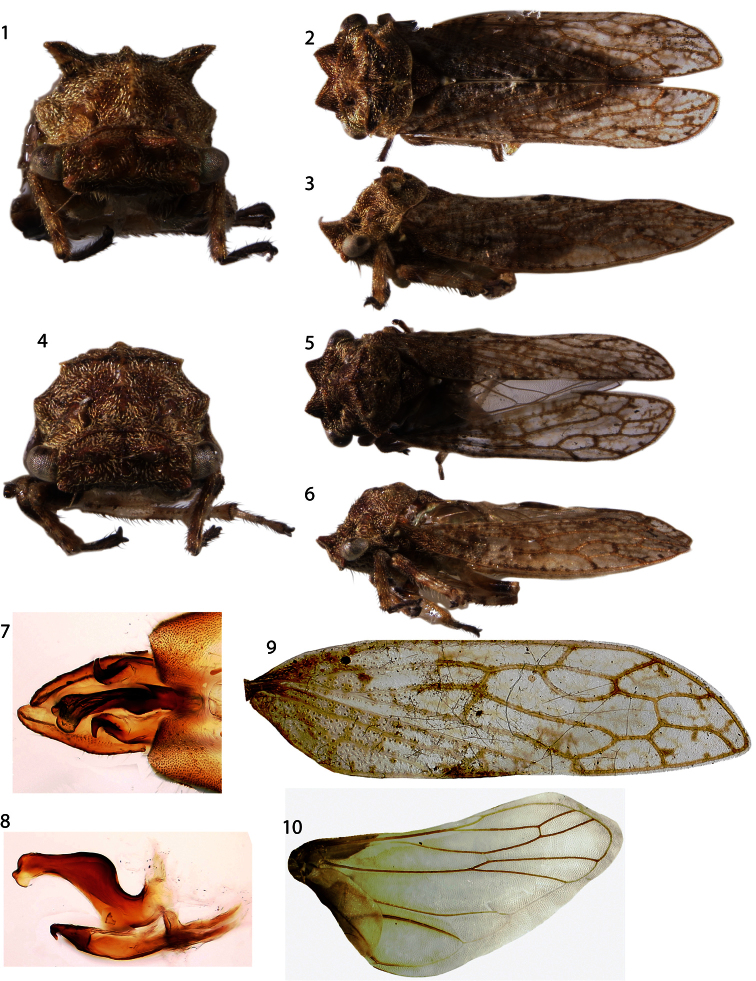
*Antillotolania myricae*, sp. n. **1–3** female habitus in anterior, dorsal, and lateral views, respectively **4–6** male, same views **7** Dorsal views, distal half of pygofer with aedeagus and styles in resting position over subgenital plates. **8** Lateral view, aedeagus and styles **9–10** left forewing and hind wing, respectively.

Female (Figs 1–3): Resembling male but pronotum with prominent suprahumeral horns, subtriangular, projecting dorsolaterally (Figs 1, 28).

**Figures 11–17.**
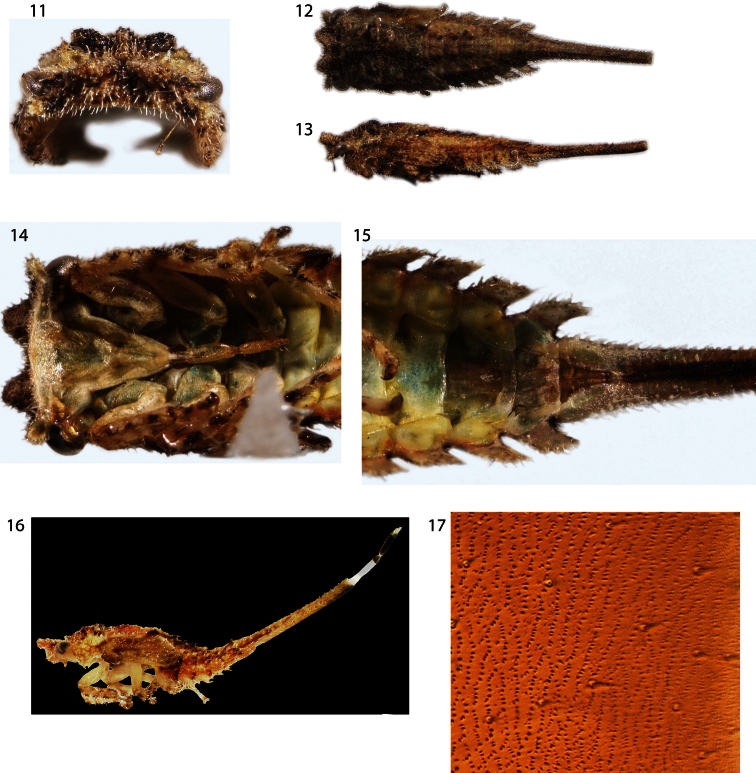
*Antillotolania myricae* sp. n. **11–15** fifth instar in anterior, dorsal, lateral and detail ventral views, respectively **16** third instar, with proportionately longer ‘anal tube’ (ventrally fused tergum IX) **17** surface vestiture of adult abdominal tergum IV.

Nymph (Figs 11–16): Fifth instar length 6.2 mm. Densely pilose and dorsoventrally compressed throughout. Head with subtriangular projections directed anteriorly as in adult, in anterior view ventral margin carinate, excavated, with ventrolateral lobes, in lateral view posterolaterally emarginate. Thoracic nota lacking scoli. Forewing ventrally emarginate. Abdominal terga IV-VII with large lateral lamellae directed posterolaterally, smaller on IV and subequal on V-VIII; tergum IX fused ventrally, forming ‘anal tube’, length subequal to remaining terga combined in last instar, as long as remainder of abdomen and thorax combined in earlier instars. Terga III-VIII with 2 pairs of enlarged chalazae, the first near mid line and the second between the first and the abdominal lamellae. Tergum IX slightly wider at base, otherwise parallel-sided, completely fused ventrally. Nascent genitalia barely exceeding posterior limit of tergum VIII lamellae (Fig. 15).

##### Material examined.

Holotype male (USNM), Puerto Rico, municipio Maricao, km 63.1 rt. 120, ca. 4 air km S Maricao. 18°08.429N; 66°58.322W, 777m, 2 May 2005. S. McKamey & B. V. Brodbeck. Paratypes (USNM, NCSU): 3 females, 11 nymphs, same locality as holotype. 3 early instar, mun. Patillas-San Lorenzo, km 5 rt.7740 nr. jxn. rt. 181, 18.10002°N; 66.01812°W, 664 m, 27 Feb 2007, S. McKamey & L.L. Deitz. 2 males, 2 females, 3 nymphs, mun. Guayama, km. 0.7 rt. 742 off rt. 7741 nr. El Chino, ca. 6 air km N Guayama, 18.05422°N; 66.10001°W, 632 m, 27 Feb 2007, S. McKamey & L.L. Deitz. 2 females, 1 nymph, mun. Cayey, rt. 7737, 1.5 air km SE Cayey, 18.08516°N; 66.17194°W, 730 m, 27 Feb 2007, S. McKamey & L.L. Deitz. 1 male, 2 nymph, mun. Cayey, rt. 184 just S jxn. 173, nr. Carite Recreational Area, 18.13181°N; 66.07427°W, 497 m, 2 March 2007, S. McKamey.

##### Host.

All specimens collected from *Myrica splendens* (Sw.) DC., Myrtaceae, a weedy species of the West Indies, Mexico, Central and South America.

##### Habitat.

Moist highlands of Puerto Rico.

##### Remarks.

Based on our series of 11 adults and over 20 immatures, the venation and nymphal characters coded ambiguously in phylogenetic estimates of the family have been determined, as discussed below. No adults of the new species were obtained from rearing, but both adults and nymphs were repeatedly obtained from the same host at the same time, at a variety of localities, without finding any other membracids. Note that the male of *Antillotolania extrema*, if discovered, may have smaller suprahumeral horns, as evidenced by the strong sexual dimorphism exhibited by the new species. The first couplet in the key provided by Cryan and Bartlett (2002) divides species by the presence or absence of developed suprahumeral horns, hence the males and females of the new species would key out separately. The following table enables identification of adults of all species in the genus.

Characters and states:

1. Suprahumeral horns present only as carinae (0) or projecting from adjacent pronotal surface (1).

2. Forewing vein R_4+5_ fused with R_1_ basad (0) or distad (1) of fork of vein M.

3. Forewing crossvein m-cu3 originating basad (0) or distad (1) of fork of vein M.

4. Forewing vein A_1_ smoothly merging with claval vein (0) or bent at a right angle and perpendicularly connecting to clavela vein (1).

5. Metathoracic tibia with cucullate setae in rows I, II, and III (0) or row II only (1).

### *Deiroderes* Ramos

*Deiroderes* contains three species: *Deiroderes inermis* Ramos from Puerto Rico and nearby islands of the British West Indies, *Deiroderes inornatus* Cryan & Deitz from Jamaica, and *Deiroderes punctatus* Metcalf & Bruner from Cuba. These were represented by a total of 13 specimens, with nothing known of their biology except one host record for *Deiroderes inermis*: *Capparis indica* (L.) Fawc. & Rendle (Capparaceae) (but see Cryan & Deitz [2002] regarding a conflict with this host record).

#### Deiroderes inermis

Ramos

http://species-id.net/wiki/Deiroderes_inermis

Figs 18–27

##### Description.

**Figures 18–27.**
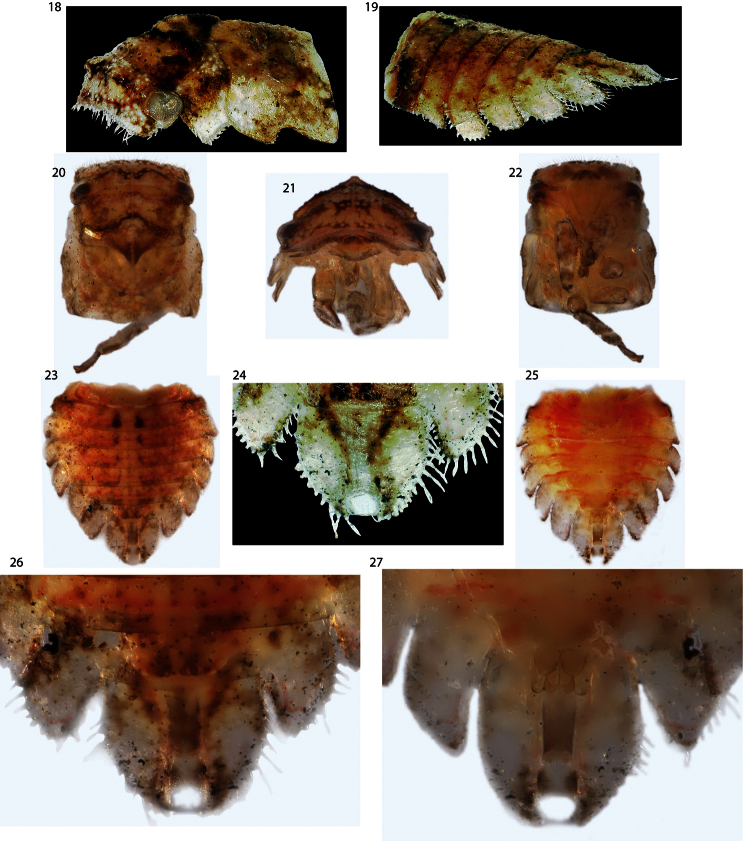
*Deiroderes inermis* immature, bisected during capture. **18, 20–22** head and thorax in oblique, dorsal, anterior, and ventral views, respectively **19, 23–27** abdomen in oblique (**19**), dorsal (**23, 24, 26**) and ventral (**25, 27**) views. Head processes visible in **18**, anal tube opening visible in **24**, and nascent genitalia visible in **27**.

Nymph (fifth instar): Length 3.5 mm. Glabrous throughout. Head with small protrusions (Fig. 18) in same placement as the large subtrianglar projections of *Antillotolania*, in anterior view ventral margin carinate, head ventrally excavated, with foliaceous ventrolateral lobes (Figs 20, 21), in dorsal view quadrate, in lateral view not emarginate. Thoracic nota lacking scoli. Forewing emarginate. Abdominal terga IV–VII with large lateral lamellae, directed posterolaterally, smallest on tergum IV and increasing in size posteriorly; tergum IX fused ventrally, forming short ‘anal tube’, length about 2 × longer than tergum VIII. Terga III–VIII each with 1 pair of enlarged chalazae near mid line. Tergum IX dorsoventrally compressed. Nascent genitalia barely exceeding posterior limit of tergum VII lamella.

**Figures 28–32.**
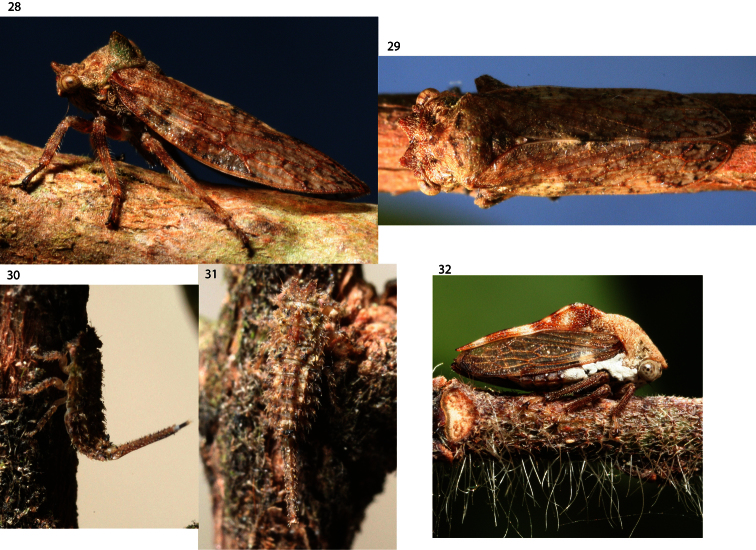
Photographs of live Puerto Rican Membracidae. **28–31** *Antillotolania myricae*, sp. n. adult female (**28**), adult male (**29**), third instar (defensive position) (**30**), and fifth instar (**31**), all on *Myrica splendens* (Myrtaceae) **32** *Nessorhinus abbreviatus* Ramos in same xeric habitat of *Deiroderes inermis* type locality (Guánica) on an unidentified host.

##### Remarks.

Caught sweeping a uniform stand of *Capparis indica*, a recorded host of *Deiroderes inermis*, adjacent to Guánica State Forest, which is the type locality of *Deiroderes inermis*. The Guánica area of Puerto Rico is arid and other membracids were previously unknown from there until L.L. Deitz (North Carolina State University) and SHM discovered *Nessorhinus abbreviatus* Ramos (Fig. 32) in February, 2007, on a different, unidentified host in February, 2007. Nymphs of *Nessorhinus gibberulus* Stål are known (McKamey unpubl.) and the fifth instars are several millimeters longer than that of *Deiroderes inermis*.

## Discussion

The anal tube (a ventrally fused abdominal segment IX, from which the anal segments protrude when defecating) present in nymphs of both *Antillotolania* and *Deiroderes* is a diagnostic feature of Membracidae. The nymphs of both genera display many features characteristic of other cryptic membracid nymphs: a flattened body and large abdominal lamellae that break up their body outline and an emarginate forewing pad providing a space for the mesothoracic tibia to rest, increasing their crypsis, suggesting that the two genera are correctly placed in that family. In some other membracid immatures with emarginate forewing pads, the tibiae are also flattened, but this is not the case in *Antillotolania* or *Deiroderes*. Instead, these have a pronotum that is posterolaterally emarginate, providing a resting place for the prothoracic tibia as well, as also occurs in some other membracids, such as some Darninae.

Placing *Antillotolania* and *Deiroderes* to subfamily and tribe is more problematic. The two possible subfamilies (with short pronotum) are Nicomiinae and Stegaspidinae. There are few nicomiine immatures known and all have been associated indirectly with adults due to the solitary nature of the species and difficulty of rearing adults. An illustration of a *Tolania* Stål nymph, which lacks any trace of abdominal lamellae, was provided by Dietrich et al. (2001). Thus, as far as known, nicomiine immatures lack abdominal lamellae. In contrast, all stegaspidines whose immatures are known, encompassing both Stegaspidini and Microcentrini, have well developed abdominal lamellae (Cryan and Deitz 1999a, 1999b, 2000; Cryan et al. 2003). The presence of foliaceous ventrolateral lobes on the head of both *Antillotolania* and *Deiroderes* also allies them with Stegaspidinae. The surface vestiture of the adult abdomen in *Antillotolania* and *Deiroderes* is shared with *Microcentrus*. This feature should not be construed as additional supporting evidence for their inclusion in Microcentrini or even Stegaspidinae because, firstly, other Membracoidea inside and outside the family Membracidae have the same character state and, secondly, the vestiture of *Antillotolania* and nicomiids were not examined in Dietrich’s (1989) survey. The only known membracid nymphs with elongate, ventrally fused ‘anal tubes’ (Figs 12–16) are *Tolania* and *Antillotolania*, suggesting that this feature may be a synapomorphy and thus evidence of a nicomiine relationship.

In a phylogenetic study, Dietrich et al. (2001) recovered *Deiroderes* in Stegaspidinae and correctly predicted character states of the immatures, including the synapomorphy of the subfamily (head with foliaceous ventrolateral lobes, Figs 18, 20–22). In contrast, *Antillotolania* was recovered as a sister-group to [Nicomiinae + Centronodinae], but the analysis incorrectly predicted several character states: the anal tube is cylindrical, not ventrolaterally angulate, there are two rows, not one, of enlarged chalazae on each side of the abdomen (Fig. 12) and the head has foliaceous ventrolateral lobes (Figs 11, 14) again, a synapomorphy of Stegaspidinae). Based on these findings the subfamily placement of the two genera treated here remains unclear.

It may be that the head processes and emarginate forewing pads (Figs 13, 16) found in *Antillotolania*, *Deiroderes*, and some other cryptic membracids (but not in stegaspidine immatures) are homologous, giving morphological support to the hypothesis of Cryan et al. (2000) that these two enigmatic Antillean genera are sister-taxa.

## Supplementary Material

XML Treatment for Antillotolania myricae

XML Treatment for Deiroderes inermis
